# Comparative Analysis of Primers Used for 16S rRNA Gene Sequencing in Oral Microbiome Studies

**DOI:** 10.3390/mps6040071

**Published:** 2023-08-06

**Authors:** Hee Sam Na, Yuri Song, Yeuni Yu, Jin Chung

**Affiliations:** 1 Department of Oral Microbiology, School of Dentistry, Pusan National University, Yangsan 50612, Republic of Korealuckcute@naver.com (Y.S.); 2 Oral Genomics Research Center, Pusan National University, Yangsan 50612, Republic of Korea; 3 Dental Research Institute, BK21 PLUS Project, School of Dentistry, Pusan National University, Yangsan 50612, Republic of Korea; 4 Interdisciplinary Program of Genomic Science, Pusan National University, Yangsan 50612, Republic of Korea; 5 Department of Biomedical Informatics, School of Medicine, Pusan National University, Busan 46241, Republic of Korea

**Keywords:** 16s RNA, primer, in silico, oral microbiome, gene sequencing

## Abstract

Recent advances in genomic technologies have enabled more in-depth study of the oral microbiome. In this study, we compared the amplicons generated by primers targeting different sites of the 16S rRNA gene found in the Human Oral Microbiome Database (HOMD). Six sets of primer targeting V1–V2, V1–V3, V3–V4, V4–V5, V5–V7 and V6–V8 regions of 16S rRNA were tested via in silico simulation. Primers targeting the V1–V2, V3–V4, and V4–V5 regions generated more than 90% of the original input sequences. Primers targeting the V1–V2 and V1–V3 regions exhibited a low number of mismatches and unclassified sequences at the taxonomic level, but there were notable discrepancies at the species level. Phylogenetic tree comparisons showed primers targeting the V1–V2 and V3–V4 regions showed performances similar to primers targeting the whole 16s RNA region in terms of separating total oral microbiomes and periodontopathogens. In an analysis of clinical oral samples, V1–V2 primers showed superior performance for identifying more taxa and had better resolution sensitivity for *Streptococcus* than V3–V4 primers. In conclusion, primers targeting the V1–V2 region of 16S rRNA showed the best performance for oral microbiome studies. In addition, the study demonstrates the need for careful PCR primer selections.

## 1. Introduction

Microbiota are found in many habitats but are poorly characterized due to a high proportion of unnamed species-level taxa [1,2]. However, the human oral bacterial community is relatively well characterized at the species level and boasts many types of microbiota. In fact, the oral cavity is second only to the gut and, to date, has been reported to harbor over 700 species of bacteria [3]. Due to the various niches in the mouth, which include both hard tooth surfaces and soft oral mucosa, microbial colonization is incredibly complex [4]. Beyond its role in the initiation of the digestive process, the oral microbiome plays a crucial role in promoting oral and overall health [5]. Recent advances in genomic technologies, such as next-generation sequencing and bioinformatics, have enabled in-depth studies of the oral microbiome and revealed some of its intricacies.

The use of targeted amplification and sequencing of the 16S ribosomal RNA gene to characterize the human oral bacterial community forms the basis of the Human Oral Microbiome Database [3]. The 16S rRNA gene has nine variable regions interspersed among the highly conserved sequences [6]. However, most studies have only sequenced part of the gene due to limitations of the widely used Illumina sequencing platform, which produces short sequences at higher through-put and lower cost than the Sanger method. The selection of PCR primers for DNA amplification is a crucial preliminary step in 16S rRNA gene sequencing studies. Researchers have sequenced different regions or multiple regions of the 16S rRNA gene, including V1–V2 [7,8], V3 [9], V1–V3 [10], V3–V4 [11], V3–V5 [12], V4 [13], V5 [14], V4–V5 [15], V6–V8 [16], and V5–V7 [17], to investigate the structures of bacterial communities.

Although a small portion of the 16S rRNA gene has been considered sufficient to represent the full-length sequence in many community analyses [18], it is important that the impacts of different regions on the analytical methods commonly used in oral microbiome studies be fully understood. In this study, we examined the amplicons generated by various primers targeting different sites of the 16S rRNA genes of oral bacteria using the Human Oral Microbiome Database (HOMD) database, which is frequently employed for oral microbiome research. In addition, we compared the oral microbiomes of clinical samples using primers targeting V1–V2 and V3–V4, which were predicted to be most suitable for oral microbiome analysis.

## 2. Material and Methods

### 2.1. In Silico Evaluation of Primers and Phylogenetic Tree Construction

In silico analysis was conducted using HOMD v15.1, which contains the oral microbiome sequences of 1016 species. In silico amplicons demarcating different sub-regions of the 16S gene were generated by trimming regions defined by established primer pairs (Table 1) using Seqkit v2.1.0 [19]. Sequences predicted to be produced by each primer were aligned using MAFFT, and phylogenetic trees were constructed using *align_to_tree_mafft_fasttree* implemented in QIIME2 (version 2020.6) [20]. Constructed phylogenetic trees were visualized by using iTOL v6 [21].

### 2.2. Study Population and Plaque Sample Collection

Plaque samples were obtained from all participants at the Department of Periodontics of Pusan National University Dental Hospital (Yangsan, Republic of Korea). Buccal swab samples were obtained from the mucosa of both cheeks using a sterile dental microbrush (Safco, Buffalo Grove, IL, USA). Supragingival samples were collected by swabbing the surfaces of mesiobuccal molar sites. Participants were requested to refrain from food and oral hygiene (brushing or flossing the teeth) for 2 h before sampling, and all provided written informed consent. The study protocol was approved by the Institutional Review Board of Pusan National University Dental Hospital (PNUDH-2017-023). Samples from all subjects were stored at −80 °C until required.

### 2.3. Extraction of Genomic DNA and Next-Generation Sequencing

Total DNA was extracted from buccal and supragingival plaque using a Gram-positive DNA purification kit (Lucigen, Biosearch Technology, Novato, CA, USA) following the manufacturer’s instructions. Final DNA concentrations were measured with a NanoDrop ND-1000 spectrophotometer (Thermo Fisher Scientific, Waltham, MA, USA). Samples were stored at −80 °C until required. The V1–V2 and V3–V4 regions of the 16S ribosomal RNA gene were subjected to PCR amplifications. The primer sequences used are listed in Table 1 [22,23,24]. Amplification was performed as follows: initial denaturation for 5 min at 95 °C, followed by 30 cycles of denaturation for 30 s at 95 °C, annealing for 30 s at 55 °C, and extension for 3 min at 70 °C. Purified amplicons were combined in equimolar amounts and subjected to paired-end sequencing using HiSeq (Illumina, San Diago, CA, USA) for V1–V2 and MiSeq (Illumina, San Diago, CA, USA) for V3–V4.

### 2.4. Bioinformatic Analysis, Statistical Analysis, and Visualization

Basic microbiome analysis has been performed using QIIME2 [20] and its associated plugins. Choa1 index and Shannon’s index method were used to measure alpha diversities, and pre-trained Naive Bayes classifier, obtained using Human Oral Microbiome Database (eHOMD) 16S rRNA Extended RefSeq sequences (version 15.1) [5], were used to assign taxonomy to unique representative sequences.

## 3. Results

### 3.1. In Silico PCR Amplicon Detection and Classification

**Figure 1:**
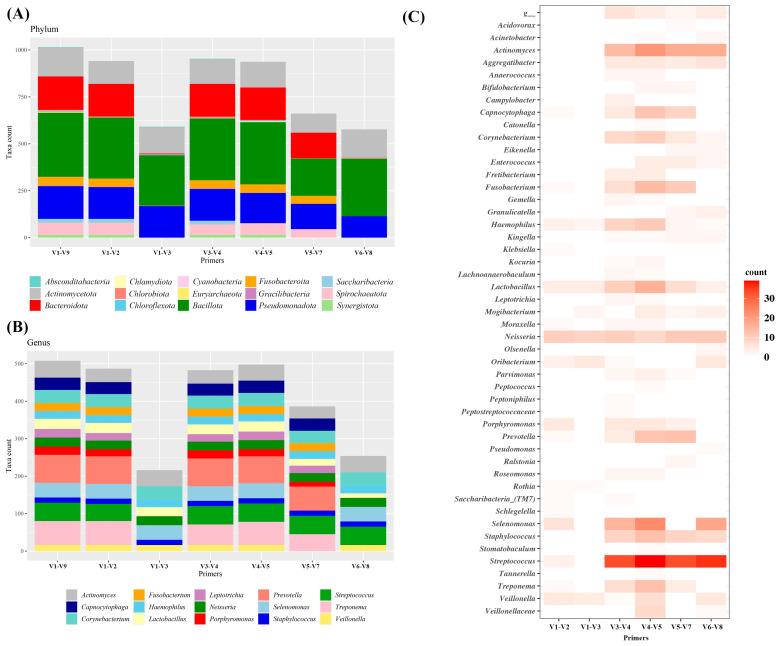
In silico generation of PCR amplicons using various primers targeting sub-regions of 16S rRNA. (**A**) Total numbers of phyla predicted to be detected by each primer, (**B**) numbers of selected genus predicted to be detected by each primer, (**C**) heatmap of unclassified or mismatch counts at the species level.

For in silico simulation, a set of full-length 16S sequences commonly used for oral microbiome studies was downloaded from a public database (eHOMD). Since these sequences incorporate PCR primer-binding sites, in silico amplicons for different sub-regions were generated using PCR primers commonly used in microbiome studies (Table 1). First, the total number of amplicons produced by each PCR primer were compared at the phylum level (Figure 1A). In the eHOMD full-length reference database, the seven most abundant phyla are Bacillota, Bacteroidota, Pseudomonadota, Actinomycetota, Spirochaetota, Fusobacteroita, and Saccharibacteria (TM7), which represent more than 96% of total sequences. In silico PCR simulation showed that primers targeting the V1–V2, V3–V4, and V4–V5 regions were able to generate more than 90% of the original input sequences. However, primers targeting the V1–V3, V5–V7, and V6–V8 regions generated less than 70% of the original input. Furthermore, primers targeting V1–V3 and V6–V8 poorly detected Bacteroidota, Spirochaetota, Fusobacteroita, Saccharibacteria (TM7), and Synergistota. Although primers targeting V4–V5 detected most of the original sequences, they failed to detect Saccharibacteria (TM7). When we examined the 15 most common genera, which accounted for over 50% of total sequences, primers targeting V1–V2, V3–V4, and V4–V5 detected more than 45% of total input. On the other hand, V5–V7 primers detected 38%, and those targeting V1–V3 and V6–V8 detected less than 25% (21% and 25%, respectively) (Figure 1B). Moreover, primers targeting V1–V3 and V6–V8 poorly detected *Prevotella, Treponema, Capnocytophaga, Leptotrichia, Porphyromonas,* and *Fusobacterium*. Taken together, primers targeting different sub-regions of 16S rRNA differed substantially in terms of the extent to which they confidently detected the oral microbiome.

To test if the amplicons were sufficient to be classified to their original taxa, the amplicon sequences generated by each primer were subjected to the Naïve Bayes classification approach implemented in QIIME2 [20]. All amplicons were correctly assigned at the family level and most at the genus level. Although there were no significant differences between amplicons assignments at higher taxonomic levels, such as genus or family, notable discrepancies were observed at the species level. Some of the amplicons produced by primers either could not be assigned or were incorrectly assigned at the species level. Primers for the V1–V2 and V1–V3 regions exhibited a low number of mismatches and unclassified sequences (48 counts and 5.1%, and 27 counts and 4.6%, respectively). However, primers targeting V3–V4, V4–V5, V5–V7, or V6–V8 regions had a high number of mismatches and unclassified sequences (175 counts and 18.4%, 255 counts and 27.2%, 149 counts and 22.5%, and 134 counts and 23.2%, respectively) (Table S1). Notably, primers targeting the V3–V4, V4–V5, V5–V7, and V6–V8 showed poor classification accuracies at the species level for microorganisms belonging to the *Actinomyces, Staphylococcus*, and *Streptococcus* genera than V1–V2 or V1–V3 primers (Figure 1C). Taken together, targeting specific sub-regions of the 16S rRNA gene with amplicons was sufficient to identify microorganisms at the genus level or higher. However, at the species level, primer selection had a significant impact on microorganism classification accuracy. While certain sub-regions (such as V1–V2 or V1–V3) represented the diversity of the 16S rRNA gene, several primers failed to identify microorganisms accurately at the species level.

### 3.2. Phylogenetic Tree Construction

**Figure 2:**
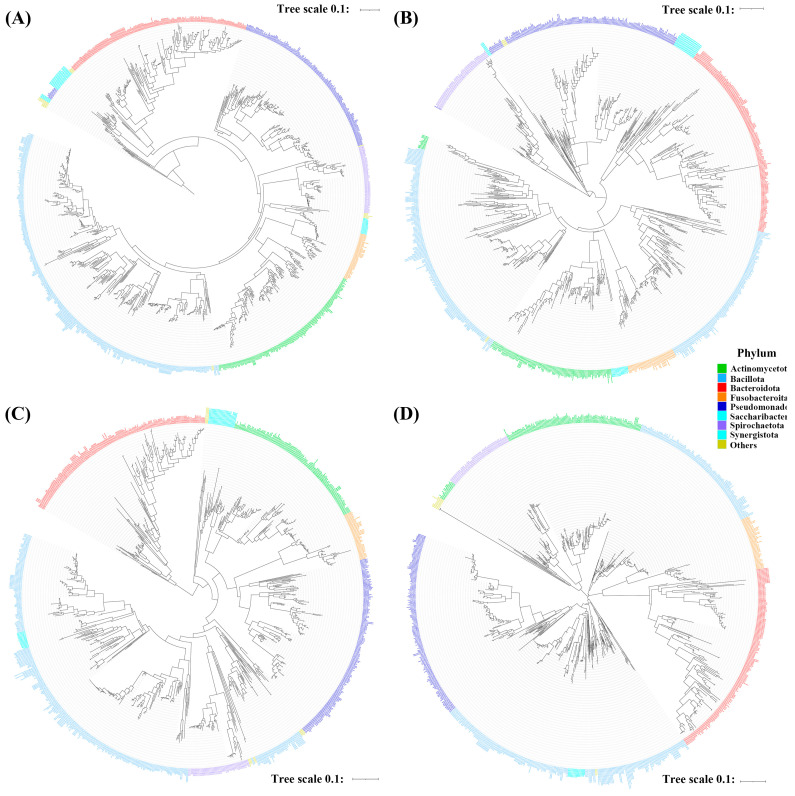
Phylogenetic trees based on the 16S rRNA gene sequence of oral microbiomes as detected by primers targeting different regions of the gene. Trees were reconstructed using the neighbor-joining method from a distance matrix constructed from aligned sequences. (**A**) V1–V9, total gene, 1343 bases, (**B**) V1–V2 region, 311 bases, (**C**) V3–V4 region, 444 bases, and (**D**) V4–V5 region, 411 bases.

**Figure 3:**
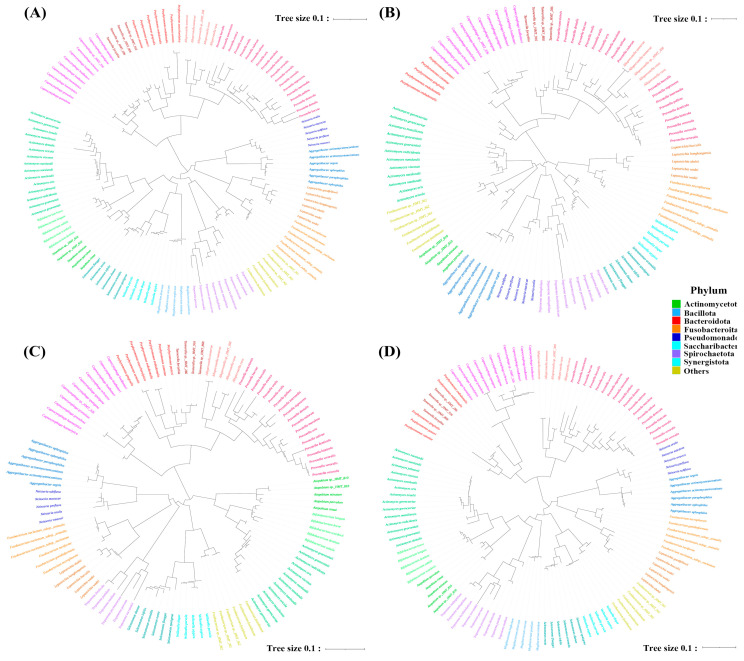
Phylogenetic trees based on the 16S rRNA gene sequences of periodontopathogens and related species as detected by primers targeting different regions of the gene. (**A**) V1–V9 region, (**B**) V1–V2 region, (**C**) V3–V4 region, and (**D**) V4–V5 region.

Next, the effectiveness with which different gene regions assessed distances among oral bacterial species was evaluated by constructing phylogenetic trees using amplicons obtained from primers targeting different sub-regions (V1–V2, V3–V4, V4–V5) and the total 16S rRNA region. The phylogenetic trees constructed by primers targeting V1–V2 and V3–V4 had similar structures, whereas the V4–V5 primer pair produced a tree distinct from that produced by the total 16S rRNA primer pair (Figure 2). Periodontitis is a common oral disease caused by periodontopathogens such as *P. gingivalis, T. forsythia*, and *T. denticola* [25]. Phylogenetic trees were constructed against periodontopathogens and related species to assess the efficiencies with which primers differentiated periodontopathogens. The results indicated that primers targeting the V1–V2 and V3–V4 regions generated trees closer related to those produced by total 16S rRNA primers than V4–V5 primers (Figure 3). When phylogenetic trees were constructed against *Streptococcus*, V1–V2 primers identified most streptococci to the species level, while V3–V4 and V4–V5 primers failed to discriminate *S. oralis* subspecies (Figure S1, Supplementary Materials). Overall, primers targeting V1–V2 and V3–V4 had a performance similar to that of total 16S rRNA primers in terms of differentiating periodontopathogens from the total oral microbiome.

### 3.3. Oral Microbiome Detected by V1–V2 or V3–V4 Primers

**Figure 4:**
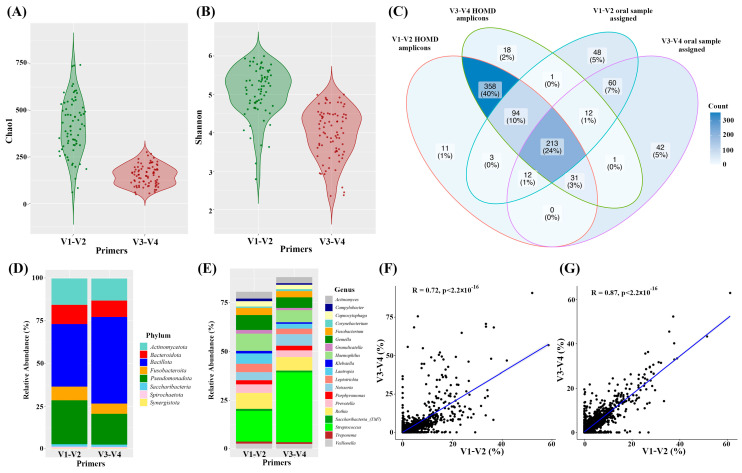
Comparison of oral microbiomes in clinical samples detected using primers targeting the V1–V2 or V3–V4 region of the 16S rRNA gene. Alpha diversity depended on the primers used. Chao1 index (**A**), Shannon index (**B**). (**C**) Venn diagram of number of in silico taxa predicted to be detected by V1–V2 or V3–V4 primers and number of species detected by each primer, (**D**) relative abundances in microbiomes detected by V1–V2 or V3–V4 primers at the phylum level, (**E**) relative abundances of the 15 most abundant taxa detected by V1–V2 or V3–V4 primers at the genus level, (**F**) correlation between relative abundances as determined by V1–V2 and V3–V4 primers at the genus level. (**G**) Correlation between relative abundance as determined by V1–V2 and V3–V4 primers at the genus level after removing the *Streptococcus* taxa count.

Following in silico simulations of oral microbiome detections, clinical samples were used to assess the performance of the V1–V2 and V3–V4 primers. In total, 70 samples from healthy adults (average age: 67 ± 8, 15 males and 22 females) were used for the clinical study (Table S2). First, Chao1 and Shannon indices were employed at the OTU level to evaluate alpha diversity. The results showed that V1–V2 amplicons had a higher Chao1 index than V3–V4 amplicons, indicating higher community richness (Figure 4A). Similarly, V1–V2 amplicons had a higher Shannon index than V3–V4 amplicons, which reflected the richness and evenness of the microbial community (Figure 4A).

After classifying taxa, species assigned by each primer and taxa predicted to be detected by the primers were compared. In terms of species detection, V1–V2 primers detected 335 species, with 108 taxa remaining unassigned at the species level. On the other hand, V3–V4 primers detected 269 species, with 102 taxa remained unassigned at the species level. Furthermore, V1–V2 primers exclusively assigned 98 species, whereas V3–V4 primers assigned only 32 species. Consequently, V1–V2 primers more effectively detected bacterial species (Figure 4B).

Next, we analyzed average relative abundances in the clinical samples. The top five phyla were Bacillota, Pseudomonadota, Actinomycetota, Bacteroidota, and Fusobacteroita. Interestingly, Bacillota had a higher relative abundance as determined by V3–V4 primers (50%) than V1–V2 primers (36.5%) (Figure 4C). At the genus level, the most prevalent genera were *Streptococcus, Haemophilus, Rothia, Gemella, Lautropia, Prevotella, Leptotrichia, Neisseria,* and *Fusobacterium*, which accounted for over 60% of the total sequences for V1–V2 primers and over 70% for V3–V4 primers. As was observed for phylum analysis, the relative abundance of *Streptococcus* was significantly higher for V3–V4 primers (35.9%) than for V1–V2 primers (15.8%) (Figure 4D).

To evaluate the influence of Bacillota and *Streptococcus* on the relative abundances of other taxa, the correlation between V1–V2 and V3–V4 was analyzed after removing *Streptococcus* at the genus level. The correlation coefficient between V1–V2 and V3–V4 at the genus level was 0.72 when all taxa were evaluated (Figure 4F), but when the *Streptococcus* counts were excluded and relative abundances were recalculated, the correlation coefficient increased to 0.87 (Figure 4G). Taken together, the relative abundance of *Streptococcus* was significantly higher when V3–V4 primers were used for the oral microbiome study, and the relative abundances of other taxa, as determined using V1–V2 and V3–V4 primers, were similar.

**Figure 5:**
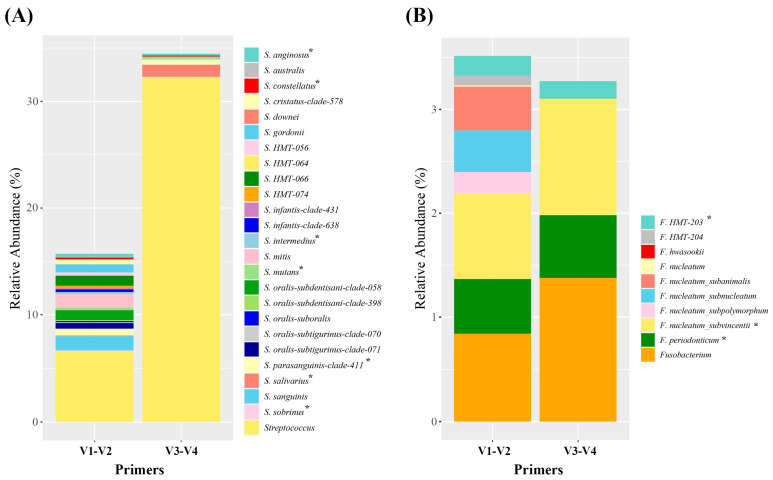
Relative abundances in clinical samples at the species level, as determined using detected V1–V2 primer or V3–V4 primers. (**A**) Relative abundances of Streptococcus species. (**B**) Relative abundances of Fusobacterium species. * indicates species identified by both V1–V2 and V3–V4 primers.

At the species level, the relative abundances of *Streptococcus* and *Fusobacterium* differed for V1–V2 and V3–V4 primers. When *Streptococcus* was analyzed, V1–V2 primers identified 15.7% of taxa as *Streptococcus,* and 24 taxa were assigned to species (9.15%). In contrast, V3–V4 primers identified 34.5% of taxa as *Streptococcus,* only assigning eight taxa to the species level (2.22%), and assigned the majority of taxa to the genus level (32.2%). Especially, V3–V4 primers failed to identify any *S. oralis* subspecies and *S. mitis*. For *Fusobacterium* analysis, both primers detected similar relative abundances at the genus level. V1–V2 primers identified nine species at the species level (2.67%), whereas V3–V4 primers assigned only three species at the species level (1.89%). V1–V2 primers distinguished four subspecies of *F. nucleatum*, whereas V3–V4 primers only detected *F. nucleatum subvincetii*. Taken together, V1–V2 primers outperformed V3–V4 primers by detecting a greater number of oral bacterial species and exhibiting higher resolutions for the identifications of *Streptococcus* and *Fusobacterium* at the species level (Figure 5).

## 4. Discussion

Comprehensively cataloging the diversity of bacteria and archaea present in the human mouth is crucial for establishing associations between specific taxa and healthy and disease states, as the oral microbiome plays a vital role in promoting both oral and overall health [5]. Characterization of the human oral bacterial community is commonly performed via targeted amplification and sequencing of the 16S ribosomal RNA gene [26,27]. However, selecting appropriate PCR primers for DNA amplification is a critical step for 16S rRNA gene sequencing studies [6], and the suitability of numerous primers targeting different regions of the 16S rRNA [7,10,11,15,16,17] gene, have been investigated. In this study, we examined the performances of various primers targeting different 16S rRNA sites to detect and determine the abundances of microbes commonly found in oral microbiomes.

First, we evaluated the performance of six commonly used bacteria-specific primers (Table 1) for short-read sequencing using in silico approaches. At the phylum level, primers designed to target the V1–V2, V3–V4, and V4–V5 regions showed high coverage, whereas primers targeting the V1–V3, V5–V7, and V6–V8 regions did not. Furthermore, primers targeting V1–V2, V3–V4, and V4–V5 provided high coverage for the 15 most commonly found genera. Thus, primers targeting different sub-regions of 16S rRNA were the 15 most commonly found to differ substantially in terms of the extent to which they could confidently detect. The V3–V4 region is one of the most widely used to profile the microbiome and provide profiles representative of diverse communities at the genus level [2,28,29,30]. V4–V5 primers have been reported to produce results that overlap poorly with other primer pairs and underrate the abundance of Bacteroidota in gut microbiome studies [31]. In conjunction with the 454 sequencing platform, which can produce a single read length of over 400 bps, the V1–V3 region was a target in the early Human Microbiome Project (HMP) [32]. As compared with the V3–V5 primer, which was the other primer target, V1–V3 had a lower OTU count in the HMP study. The V1–V2 region has been reported to capably identify most streptococci to the species level and was recommended for oral sample studies [8]. Given that amplicons contain a sub-region of 16S rRNA, we believed that it would be interesting to investigate whether amplicons can be reclassified into their original taxa. Amplicons were successfully assigned to their original taxa at a higher taxonomic level, such as genus or family, but notable discrepancies occurred at the species level. Primers designed for the V1–V2 and V1–V3 regions exhibited a low number of mismatches and unclassified sequences. Although the V3–V4 region is commonly used for profiling the microbiome, the V1–V2 region achieved comparable taxon detection and outperformed the V3–V4 region in terms of assigning amplicons to original taxa. These in silico results suggest that the V1–V2 primer pair may be more effective at detecting the oral microbiome than the commonly used V3–V4 primers.

Next, the abilities of gene regions to assess distances between oral bacterial species were evaluated by constructing phylogenetic trees. When phylogenetic trees of the total oral microbiome were constructed, primers targeting V1–V2 and V3–V4 produced trees with structures similar to primers targeting the full 16S rRNA region (Figure 2). *Streptococcus* is one of the most commonly found bacteria in the oral cavity and includes a large number of species. Cabral et al. reported that the V4 and V3–V4 regions have limited ability to differentiate oral streptococci, whereas the V1–V2 region can successfully identify most *Streptococcal* species [8]. Moreover, the V1–V2 region produced clustering of closely related species similar to that produced by full 16S rRNA, whereas the V3–V4 region failed to do so [33]. We also observed similar results when *Streptococcus* was analyzed (Figure S1). Periodontitis is one of the most common oral diseases caused by periodontopathogens, such as *P. gingivalis, T. forsythia*, and *T. denticola* [25]. Thus, we constructed phylogenetic trees for periodontopathogens and related species. V1–V2 and V3–V4 regions generated trees relatively similar to those of full 16S rRNA (Figure 3), suggesting that in silico, V1–V2 primers are comparable to V3–V4 primers in terms of clustering the total oral microbiome and periodontopathogens and that V1–V2 primers may perform better *Streptocococcus* clustering.

Finally, the performances of V1–V2 and V3–V4 primers were assessed using clinical oral samples. Chao1 and Shannon indices indicated that V1–V2 primers outperformed V3–V4 primers by detecting a greater number of species. When the total numbers of assigned taxa for each primer were compared, V1–V2 primers were more effective at detecting bacterial species in clinical oral samples. Due to amplicon size (Table 1), samples were subjected to paired-end sequencing using HiSeq for V1–V2 and MiSeq for V3–V4 for NGS analysis. It is well known that forward reads consistently exhibit high sequencing quality using HiSeq and MiSeq, whereas reverse reads tend to display lower quality towards the distal end using MiSeq [34]. The occurrence of low-quality bases towards the distal end of a sequence can impede the joining step, result in an inability to join, and ultimately lead to read losses during analysis [35]. Thus, sequencing platform differences may contribute to alpha diversity differences.

Analysis of average relative abundances revealed that the abundance of Bacillota was higher for V3–V4 primers (50%) than for V1–V2 primers (36.5%). Similarly, the relative abundance of *Streptococcus* was significantly higher for V3–V4 primers (35.9%) than for V1–V2 primers (15.8%). Since *Streptococcus* is one of the most prevalent bacteria in the oral cavity [25], its abundance should have a significant effect on the abundance of other species. When *Streptococcus* was removed before comparing the abundance of other bacteria, the abundances of the other genera were similar, which suggests that primers exert a significant selective influence on particular species, and if these species are abundant within a community, it might impact the overall abundance of other bacteria.

Analysis at the species level revealed differences in the relative abundances of *Streptococcus* and *Fusobacterium*. V1–V2 primers successfully assigned 58% of *Streptococcus* to 24 species, leaving 41% identified to the genus level, whereas V3–V4 primers only assigned 9.5% of *Streptococcus* to eight species and identified the majority of taxa at the genus level (93.5%). In silico, *Streptococcus* phylogenetic trees showed that V1–V2 primers could identify most streptococci to the species level, while V3–V4 primers failed to differentiate *S. oralis* and *S. mitis* (Figure S1). Taken together, clinical sample and in silico results concurred, indicating that V3–V4 primers offer a limited resolution for the identification of *Streptococcus*. In the case of *Fusobacterium*, V1–V2 and V3–V4 primers detected similar relative abundances at the genus level (around 3%), and V1–V2 primers identified nine species (2.67%), while V3–V4 primers identified three species (1.89%). Furthermore, V1–V2 primers were able to distinguish four subspecies of *F. nucleatum*, whereas V3–V4 primers only detected *F. nucleatum subvincetii.* Overall, these results suggest that the V1–V2 primers are more accurate than V3–V4 primers for identifying *Streptococcus* and *Fusobacterium* at the species level in oral microbiome studies.

The study results are conclusive, but there are several limitations. First, the number of clinical samples used in this study was relatively small. Thus well-designed, larger-scale studies are required to validate our results. In addition, various sampling sites, age groups, ethnic backgrounds and clinical diseases should be included in a future study. Second, the abundances of *Streptococcus* and other species of interest were not quantified, and thus, we suggest that in future studies, this be undertaken by qPCR. Finally, since targeting specific regions of 16S rRNA can cause taxonomic classification ambiguities, we suggest that combining this with long-read sequencing should enable more accurate and sensitive identification of bacteria [6].

To conclude, our findings have significant implications for oral microbiome studies. In particular, they emphasize the importance of selecting PCR primers carefully. Furthermore, they show that the primary advantage of using the V1–V2 primer set is its superior ability to identify more taxa and its higher resolution for *Streptococcus* species. Since *Streptococcus* constitutes a significant proportion of oral microbial communities, our findings suggest that the V1–V2 primer set should be preferred when investigating microbial community dynamics in the oral environment.

## Figures and Tables

**Table 1:** Primers used to target the 16S rRNA subregion.

Region	Forward	Reverse	Size (bp)
V1–V2	AGAGTTTGATYMTGGCTCAG	TGCTGCCTCCCGTAGRAGT	311
V1–V3	TNANACATGCAAGTCGRRCG	WTTACCGCGGCTGCTGG	450
V3–V4	CCTACGGGNGGCWGCAG	GACTACHVGGGTATCTAATCC	444
V4–V5	GTGYCAGCMGCCGCGGTAA	CCGYCAATTYMTTTRAGTTT	411
V5–V7	AACMGGATTAGATACCCKG	ACGTCATCCCCACCTTCC	394
V6–V8	CAACGCGAAGAACCTTACC	GACGGGCGGTGWGTRCA	424

## Data Availability

The raw sequencing data have been deposited in NCBI GenBank under BioProject ID PRJEB63675.

## Supplementary Materials

The following supporting information can be downloaded at: https://www.mdpi.com/article/10.3390/mps6040071/s1, Figure S1: Phylogenetic trees based on 16S rRNA gene sequence of *Streptococcus* as detected by various primers targeting different regions of the gene, Table S1: Summary of amplicon classification, Table S2: Characterization of clinical samples.
