# Supplementary material for: Comparative Analysis of Primers Used for 16S rRNA Gene Sequencing in Oral Microbiome Studies
Source: Methods Protoc. 2023 Aug 6;6(4):71. doi: 10.3390/mps6040071 (PMC10460062; doi:10.3390/mps6040071)
Supplement: Supplementary file 1 [file mps-06-00071-s001.zip › Supplementary Table.pdf]

Supplementary Table

Supplementary Table S1. Summary of amplicon classification

	Unclassified amplicons	Unclassified %	Mismatch	Mismatch %
V1-V2	48	5.10	1	0.11
V1-V3	27	4.57	1	0.17
V3-V4	175	18.36	8	0.84
V4-V5	255	27.21	13	1.39
V5-V7	149	22.54	8	1.21
V6-V8	134	23.22	2	0.35

Supplementary Table S2. Characterization of clinical samples

Sex	Age	Sampling Site
Male (n=15)	67.7 ± 8.3	Buccal (n=35)
Female (n=22)	67.8 ± 8.0	Supragingival (n=35)
