# Supplementary figures and images for: Comparative Analysis of Primers Used for 16S rRNA Gene Sequencing in Oral Microbiome Studies
Source: Methods Protoc. 2023 Aug 6;6(4):71. doi: 10.3390/mps6040071 (PMC10460062; doi:10.3390/mps6040071)

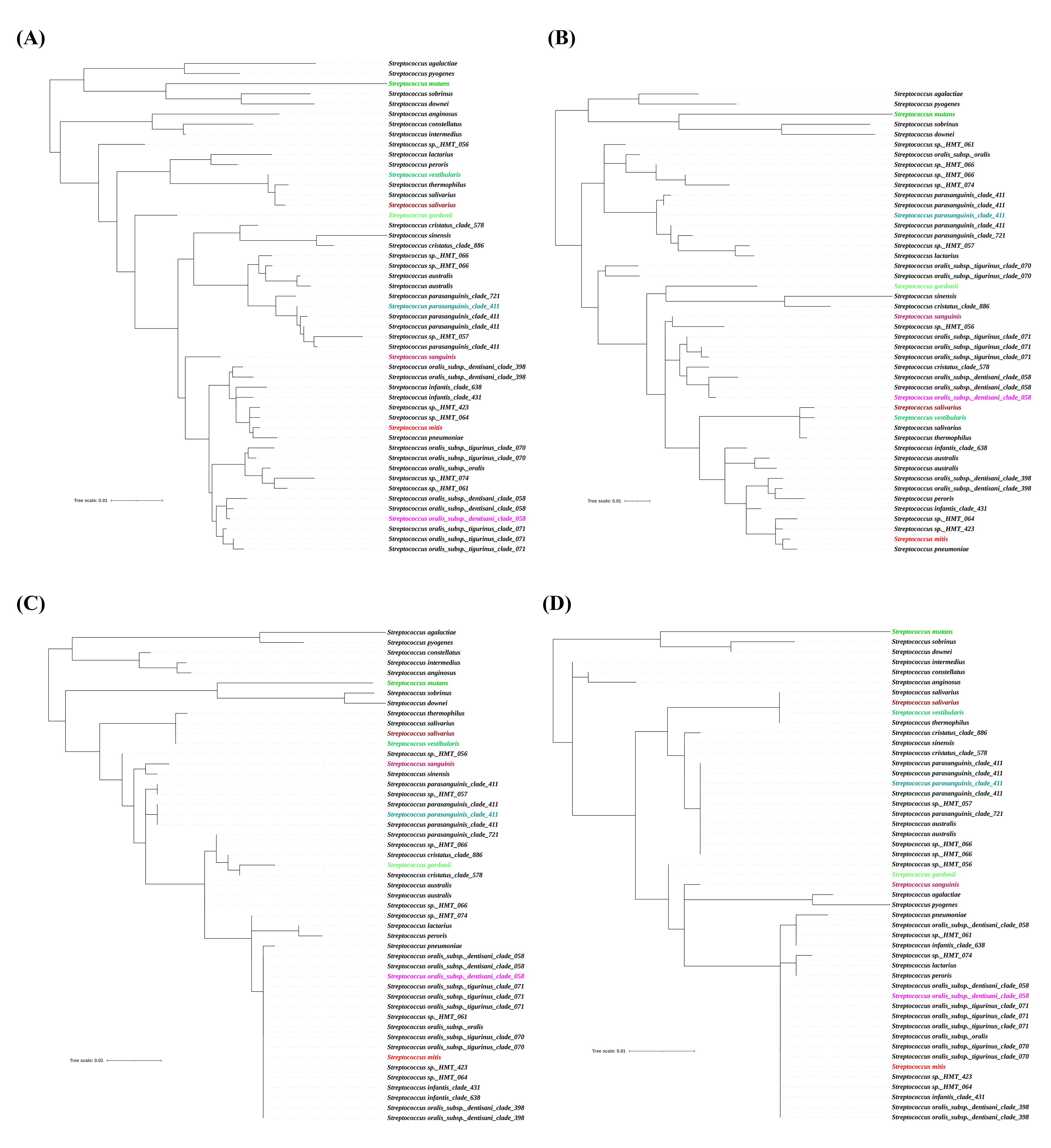

Supplement: Supplementary file 1 [file mps-06-00071-s001.zip › FigS1.tif]
